# Major Septal Defects: Comparative Study of Down Syndrome and Non-Down Syndrome Infants, Before and After Surgery

**DOI:** 10.12669/pjms.36.5.1743

**Published:** 2020

**Authors:** Saleha Aziz, Maaha Ayub, Laiba Masood, Muneer Amanullah, Rukhsana Hameed, Shiraz Hashmi, Waris Ahmad

**Affiliations:** 1 Saleha Aziz Medical Student, Aga Khan University, Karachi, Pakistan; 2 Maaha Ayub Medical Student, Aga Khan University, Karachi, Pakistan; 3 Laiba Masood Medical Student, Aga Khan University, Karachi, Pakistan; 4 Prof. Dr. Muneer Amanullah Professor of Pediatric Cardiothoracic Surgery, NICVD; Karachi, Pakistan; 5 Dr. Rukhsana Hameed Head of Department, Maternal and Child Health, Institute of Public Health, Lahore, Pakistan.; 6 Shiraz Hashmi Analyst, Cardiothoracic Surgery, Aga Khan University, Karachi, Pakistan; 7 Dr. Waris Ahmad Assistant Professor, Pediatric Cardiothoracic Surgery, Aga Khan University, Karachi, Pakistan

**Keywords:** Down Syndrome, Septal Defects, Atrioventricular, Inlet VSD, AVSD, Endocardial Cushion Defect

## Abstract

**Objective:**

To compare pre-operative, intra-operative, and post-operative parameters in Down syndrome (DS) and non-DS patients with atrioventricular septal defects (AVSD) and inlet ventricular septal defects (VSD) in a tertiary care hospital in Pakistan.

**Methods:**

We conducted a retrospective study at Aga Khan University, Pakistan. All complete atrioventricular septal defect (CAVSD), partial atrioventricular septal defect (PAVSD), and VSD with inlet extension surgical cases from January 2007 to January 2019 were included. Patients with congenital heart diseases other than those listed above were excluded.

**Results:**

In 61 cases, 18 had DS. Median age, mean body surface area (BSA), and height were lower in DS patients compared to non-DS patients: 7.0 vs 23.0 months, 0.311 vs 0.487 m2, and 63 vs 82 cm, respectively. Bypass duration, aortic cross clamp time, post-operative ventilator hours, dose of inotropes, CICU stay, and total hospital stay were all significantly higher in the DS group. The odds ratio (955% CI) for mortality in DS babies was 6.2 (1.4, 27.1), p=0.015, after adjusting for age, weight, and height. The overall morbidity was comparable between the two groups, demonstrating no significant difference after adjusting for confounders.

**Conclusion:**

DS babies with AVSD and inlet VSD are at a greater risk of mortality compared to non-DS babies, particularly those with CAVSD. Furthermore, DS babies undergo surgery at a younger age and require more aggressive post-operative therapy and monitoring due to the development of complications.

## INTRODUCTION

The most common congenital heart defects in Down syndrome (DS) babies are atrioventricular septal defects (AVSD) and ventricular septal defects (VSD) with a prevalence of 54% and 40%, respectively.^1^ Numerous reports of association of inlet VSD with DS babies are also present as they are often found as a part of the AVSD complex.^2^ These septal defects typically present in the fetus or neonate and are a significant cause for cardiac morbidity and mortality in this age group, especially in complete atrioventricular septal defects (CAVSD).^3^

Over the years, there has been a decline in the mortality and reoperation rates among patients undergoing surgical treatment for AVSD indicating overall improved outcomes due to better surgical technique and peri-operative care.^4^,^5^ However, there is a reported discrepancy in the clinical presentations and treatment outcomes of DS and non-DS patients with AVSD. Differences in treatment outcomes are also present among the different types of the major congenital septal defects. Patients suffering from DS and CAVSD concurrently have a higher mortality risk compared to patients without these conditions; the early postoperative morbidity rate among patients with DS and AVSD is also higher.^6^ Considering the differences in various parameters among DS and non-DS patients with septal defects, it is important to assess and compare the pre-operative, intra-operative and post-operative parameters of the two groups in loco-regional settings.

## METHODS

We conducted a retrospective chart review at Aga Khan University Hospital, Karachi, Pakistan from January 2007 to January 2019 and found 61 cases that satisfied our inclusion criteria. All CAVSD, PAVSD, and VSD with inlet extension surgical cases were included. Any other congenital heart disease or concomitant congenital heart diseases (two or more pathologies) were excluded. When data sets were normally distributed, we used mean and standard deviation as the measurements of central tendency, and independent sample t-test for comparing means of continuous variables between discrete data for two groups and one-way ANOVA test for more than two groups. In cases of non-normally distributed data, median and inter-quartile ranges were applied. For non-parametric data Mann Whitney U test and Kruskal Wallis test were applied. The chi-squared test was used for the comparison of qualitative data. A multivariate logistic regression model was built to adjust for confounders. A p-value <0.05 was considered significant. All statistical analysis was performed using SPSS version 20.0 (IBM Corp., Armonk, N.Y., USA).

## RESULTS

**Fig. 1:**
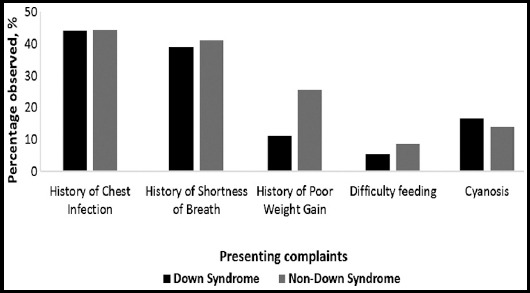
Comparison (proportion) of presenting complaints in Down vs. non-Down syndrome patients.

Our study included 61 patients, of which there were 32 male and 29 female babies. Amongst this population, 30% patients had DS. Descriptive analysis shows that patients with DS underwent surgery at a younger age, and had lower body surface areas and heights (Table-I). Comparisons of the common presenting complaints among the groups are shown in Fig.1.

**Table-I:** Pre, intra, and post-operative parameters in Down and non-Down syndrome.

	With Down Syndrome (n=18)	Without Down Syndrome (n=43)	p-value
Gender, n (%)			0.381
Male	11 (61%)	21 (49%)	
Female	7 (39%)	22 (51%)	
Age at surgery, median (IQR), months	7 (5-19)	23 (5-78)	0.028
Weight, median (IQR), kg	6 (4-8)	9 (5-16)	0.067
Height, median (IQR), cm	63 (59-76)	82 (65-114)	0.011
Body surface area, median (IQR), 10^-2^m²	31.1 (26.7-41.9)	48.7 (29.5-77.7)	0.037
Bypass time, median (IQR), minutes	135 (95-185)	108 (80-130)	0.024
Aortic cross clamp time, mean (SD), min	105.3 (39.3)	77.5 (41.8)	0.019
Minimum temperature, mean (SD), °C	32.1 (1.6)	32.4 (1.9)	0.489
Mortality, n (%)	9 (50%)	4 (9%)	0.001
CAVSD n (%)	8 (66.7%)	0 (0%)	
VSD n (%)	1 (25%)	4 (23.5%)	
PAVSD n (%)	0 (0%)	0 (0%)	
Re-opening, n (%)	2 (11%)	1 (2%)	0.205
Ventilator support, n (%)	17 (94%)	32 (74%)	0.089
Ventilation hours, median (IQR), hours	96.5 (45.3-202.8)	10.0 (2.0-36.0)	<0.001
Inotrope administration, n (%)	17 (94)	35 (81)	0.259
Inotrope dose, median (IQR), mcg/kg/min	6.5 (1.0-16.3)	3.0 (0.0-5.0)	0.026
CICU stay, median (IQR), days	9 (5-16)	6 (3-8)	0.042
Total hospital stay, median (IQR), days	11 (8-22)	8 (7-15)	0.033

Intra-operatively, the bypass time and aortic cross clamp time were both significantly higher in the DS group (p<0.05). Post-operatively, the hours of ventilator support, the administered dose of inotropes, along with the total CICU and hospital stay were all significantly greater in DS patients (p<0.05). Some of the commonly observed post-operative morbidities included arrhythmia, pleural effusion, pneumothorax, pulmonary hypertension, and sepsis which were more common in DS than non-DS patients. Other morbidities, including pericardial effusion and chest infections, were more common in the non-DS group. Both pleural and pericardial effusions were managed conservatively as well as via chest tube insertion, while pneumothorax was primarily managed via tube thoracostomy. Patient characteristics for pre-, intra-, and post-operative parameters in DS and non-DS patients are presented in Table-I.

The most prevalent defect was CAVSD, followed by VSD and PAVSD. DS patients had a greater prevalence of CAVSD compared to non-DS patients (67% vs. 38%), whereas VSD was comparable (22% vs. 24%) and PAVSD was higher in non-DS patients (11% vs. 24%).

Overall operative mortality was 21.3%, however, DS had a greater number of deaths as compared to non-DS [n (%): 9 (50%) vs. 4 (9%)]. Furthermore, mortality was significantly associated with lower age (p<0.001), height (p=0.004), weight (p=0.002) and body surface area (p=0.002). The association of mortality and DS still persists after adjusting for age, height, and weight [(OR, 95% CI) 6.2 (1.4, 27.1) p=0.015] (Table-II). Mortality rate of CAVSD patients in the DS group vs non-DS group was 66.7% vs. 0% (Table-I). Moreover, the patients that did not survive had spent a significantly greater number of hours on ventilator support (p=0.008). Common causes of mortality include cardiopulmonary arrest, sepsis, and arrhythmia.

**Table-II:** Univariate and multivariate logistic regression model for DS and non-DS.

Outcome variable	Unadjusted		Adjusted	

	OR (95% CI) Crude	P value	OR (95% CI) Adjusted*	P value
Mortality	9.8 (2.6, 38.9)	0.001	6.2 (1.4, 27.1)	0.015
Morbidity^¶^	2.0 (0.6, 6.3)	0.236	1.4 (0.4, 4.8)	0.574

* Adjusted for age, weight and height;

¶ Arrhythmia, respiratory infection, sepsis, pneumothorax, pleural and pericardial effusion, and pulmonary hypertension.

Post-operative ventilator use was associated with inotrope administration (p=0.001), specifically with higher doses of epinephrine (p=0.014) and milrinone (p=0.006) administered to patients on ventilator support. Patients on ventilator support also spent a significantly greater number of days in the CICU (p<0.001) and the hospital (p=0.002).

Patients with CAVSD and VSD were younger at the time of surgery, and had lower weight, height, and body surface area (p<0.001). Bypass and aortic cross clamp times were both highest in CAVSD patients and lowest in VSD patients (p<0.001), which suggests that CAVSD patients had more complex procedures. The minimum temperature during procedure was lowest in CAVSD patients and highest in VSD patients p=0.007 (Table-III).

**Table III:** Pre-, intra- and post-operative parameters of all patients by the type of defect.

	CAVSD (N=28)	PAVSD (N=12)	VSD (N=21)	p-value
Age at surgery, median (IQR), months	6.0 (10.5-66.8)	102.5 (60.0-156.0)	7.0 (4.0-18.5)	<0.001
Weight, median (IQR), kg	6.0 (5.0-13.9)	20.5 (13.0-43.3)	5.0 (4.0-8.0)	<0.001
Height, median (IQR), cm	67.5 (60.3-105.3)	123.5 (90.3-126.5)	102.5 (60.0-156.0)	<0.001
Body surface area, median (IQR), m²	0.3 (0.3-0.7)	0.8 (0.6-1.3)	0.3 (0.2-0.4)	<0.001
Bypass time, median (IQR), minutes	127.5 (116.0-150.0)	110.0 (92.5-150.0)	65.0 (50.0-106.0)	<0.001
Aortic cross clamp time, mean (SD), minutes	108.1 (37.7)	97.2 (39.3)	49.4 (23.1)	<0.001
Minimum temperature, mean (SD), °C	31.7 (1.8)	32.1 (1.2)	33.3 (1.8)	0.007
Mortality, n (%)	8 (29%)	0 (0%)	5 (24%)	0.122
Re-opening, n (%)	0 (0%)	1.0 (8.3%)	2.0 (10.0%)	0.259
Ventilator support, n (%)	22 (79%)	9 (75%)	18 (86%)	0.720
Ventilation hours, median (IQR), hours	29.0 (3.3-145.8)	4.0 (2.0-20.0)	20.5 (6.8-72.5)	0.160
Inotrope administration, n (%)	26 (93%)	9 (75%)	15 (71%)	0.273
Total hospital stay, median (IQR), days	9 (7-19)	7 (6-10)	10 (7-15)	0.343

## DISCUSSION

Congenital heart disease (CHD) is prevalent in 40% of patients with DS, and 0.3% in non-DS.^7^,^8^ Studies from Pakistan show that VSD and AVSD are the two most prevalent CHD in DS patients in the country, with a prevalence of 60.4% and 29.1%, respectively.^9^,^10^ However, our study shows that CAVSD has the greatest prevalence in DS patients followed by VSD: 67% and 22%, respectively. The surgical correction of CHD in these patients usually involves greater risk of postoperative complications and mortality.^11^

A study done in the United States on mortality associated with CHD from 1979 to 1997 shows increased life-expectancy of AVSD (with or without DS) and VSD suggesting improvements in management including surgical technique and peri-operative care.^5^ Data from Pakistan also suggests that repair of partial atrioventricular canal has good outcomes with minimal mortality and that earlier repair reduces post-operative morbidity further.^12^ Early repair of congenital heart diseases also has the benefit of less expense which is important in a low resource setting like Pakistan.^13^ Most DS patients in our study underwent repair at less than one year of age, which is consistent with other studies on AVSD repairs in DS patients.^14^

The presence of DS in patients significantly increases the risk of severe morbidities that have a significant impact on the recovery period, as well as on life expectancy even after successful CHD correction.^15^ Our study showed trends of increased morbidity in the DS group, however, it did not reach statistical significance possibly due to small sample size. The mortality rate in DS patients was significantly higher than non-DS patients, even after adjustment of confounders (age, weight, height). Within this mortality group, 61.5% were diagnosed with a CAVSD. CAVSD is known to have poorer outcomes, longer median ventilation time, and intensive care unit and hospital stay (days), compared to other AVSD subtypes.^3^ However, we did not find any reported deaths in non-DS patients with CAVSD.

Interestingly, Western literature reports no significant difference or lower risk of mortality after AVSD correction in DS patients, which contrasts our findings.^6^,^16^ This raises questions of possible differences in outcomes due to genetic, racial, environmental, and social factors which have not yet been explored in this region. This also highlights the need for developing indigenous guidelines for the management of these pathologies as currently practices from other countries (particularly from Western states) are being followed in Pakistan. Local guidelines were developed in India after the National Consensus Meeting on Management of Congenital Heart Diseases, 2018, which suggested that patients in the region are often underweight, malnourished, and have comorbidities such as recurrent infections and anemia. Several patients present late with advanced level of pulmonary hypertension, ventricular dysfunction, hypoxia, and polycythemia. Therefore, the outcome after surgery in such patients has a greater possibility of being undesirable, justifying the need for revised guidelines and practices with a risk adjusted approach.^17^

DS and non-DS patients have no significant differences in preoperative hemodynamics, however, post-operative right heart pressures and pulmonary vascular resistance have remained significantly high in DS patients compared to non-DS patients.^18^ This could be a result of lung hypoplasia in DS patients, which predisposes them to pulmonary hypertension, leading to longer ventilation times and increasing postoperative morbidity. An increased cross-clamp time, as seen in our DS patient group, is also directly correlated with increased ventilation support.

Intra-operatively, DS patients in our study had a significantly higher cardiopulmonary bypass time (CBPT) and aortic cross-clamp time (XCT), with longest durations in the CAVSD group. Longer CBPT and XCT reflect more complex surgeries, indicating that DS and CAVSD patients underwent a more difficult repair.^19^

### Limitations of the study

The result of this study should be interpreted considering that this is a single center data analysis. Hence, reproducing the study on a larger scale across multiple centers of the country will yield more robust results. Since this study was conducted in one of the most urbanized areas of the country, socioeconomic parameters in the sample population may not be an accurate representation of the population at large. Furthermore, only inpatient mortality has been listed but close follow-up on the patients could shed more light on outpatient and late mortality as well.

## CONCLUSION

DS babies with AVSD and inlet VSD are at greater risk for mortality compared to non-DS babies, particularly those with CAVSD. Furthermore, DS babies undergo surgery at a younger age than non-DS babies. DS babies require more aggressive post-operative therapy and monitoring due to the development of complications.

### Authors’ Contribution

**SA:** Idea conception, data collection, manuscript writing, responsible and accountable for integrity of the work.

**MA:** Data collection, entry, and analysis.

**LM:** Data collection and manuscript writing.

**MAMA:** Supervised and encouraged medical students to pursue this research.

**RH:** Critical revision of the article.

**SH:** Data analysis and interpretation.

**WA:** Supervised the findings of this work.
